# An unusually short inter­molecular N—H⋯N hydrogen bond in crystals of the hemi-hydro­chloride salt of 1-*exo*-acetamido­pyrrolizidine

**DOI:** 10.1107/S2056989019016517

**Published:** 2020-01-01

**Authors:** Minakshi Bhardwaj, Qianxiang Ai, Sean R. Parkin, Robert B. Grossman

**Affiliations:** a University of Kentucky, Lexington, Kentucky, 40506-0055, USA

**Keywords:** crystal structure, short N—H⋯N hydrogen bond

## Abstract

The title compound [systematic name: (1R*, 8*S*)-2-acetamidoocta­hydro­pyrrol­izin-4-ium chloride–*N*-[(1*R*, 8*S*)-hexa­hydro-1*H*-pyrrolizin-2-yl)acetamide (1/1)], 2(C_9_H_16_N_2_O)·HCl or C_9_H_17_N_2_O^+^·Cl^−^·C_9_H_16_N_2_O, arose as an unexpected product when 1-*exo*-acetamido­pyrrolizidine (AcAP; C_9_H_16_N_2_O) was dissolved in CHCl_3_. Within the AcAP pyrrolizidine group, the unsubstituted five-membered ring is disordered over two orientations in a 0.897 (5):0.103 (5) ratio. Two AcAP mol­ecules related by a crystallographic twofold axis link to H^+^ and Cl^−^ ions lying on the rotation axis, thereby forming N—H⋯N and N—H⋯Cl⋯H—N hydrogen bonds. The first of these has an unusually short N⋯N separation of 2.616 (2) Å: refinement of different models against the present data set could not distinguish between a symmetrical hydrogen bond (H atom lying on the twofold axis and equidistant from the N atoms) or static or dynamic disorder models (*i.e.* N—H⋯N + N⋯H—N). Computational studies suggest that the disorder model is slightly more stable, but the energy difference is very small.

## Abstract 2

In the crystal, two AcAP mol­ecules related by a crystallographic twofold axis link to H^+^ and Cl^−^ ions lying on the rotation axis, thereby forming N—H⋯N and N—H⋯Cl⋯H—N hydrogen bonds. The first of these has an unusually short N⋯N separation of 2.616 (2) Å: refinement of different models against the present data set could not distinguish between a symmetrical hydrogen bond (H atom lying on the twofold axis and equidistant from the N atoms) or static or dynamic disorder models (*i.e.* N—H⋯N + N⋯H—N).

## Chemical context   

**Figure 1:**
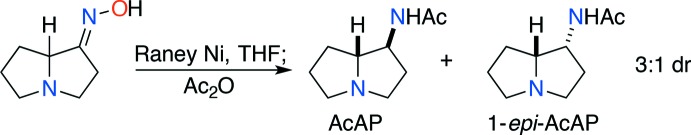
Synthesis of AcAP.

In the course of our ongoing studies on the biosynthesis of loline alkaloids (Schardl *et al.*, 2007 ▸; Pan *et al.*, 2018 ▸), we had occasion to prepare 1-*exo*-acetamido­pyrrolizidine (C_9_H_16_N_2_O; AcAP) by reduction of the corresponding oxime (Fig. 1 ▸) (Pan *et al.*, 2014 ▸). As part of our effort to prove unambiguously that the major diastereomer obtained in this reaction was indeed the *exo* diastereomer, we attempted to obtain crystals of AcAP that were suitable for X-ray analysis. We obtained sufficiently high-quality crystals by recrystallization from CHCl_3_, but to our surprise, the analysis showed that the crystals were not AcAP, but 2AcAP·HCl, with the HCl presumably originating from amine-promoted decomposition of CHCl_3_. This paper describes the structure of crystalline 2AcAP·HCl, which features an unusually short ^+^N—H⋯N inter­action.
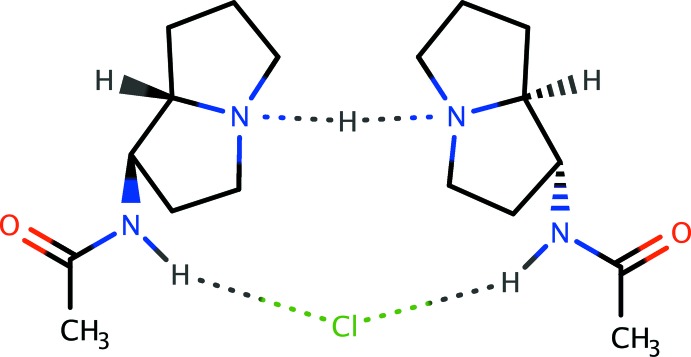


## Structural commentary   

**Figure 2:**
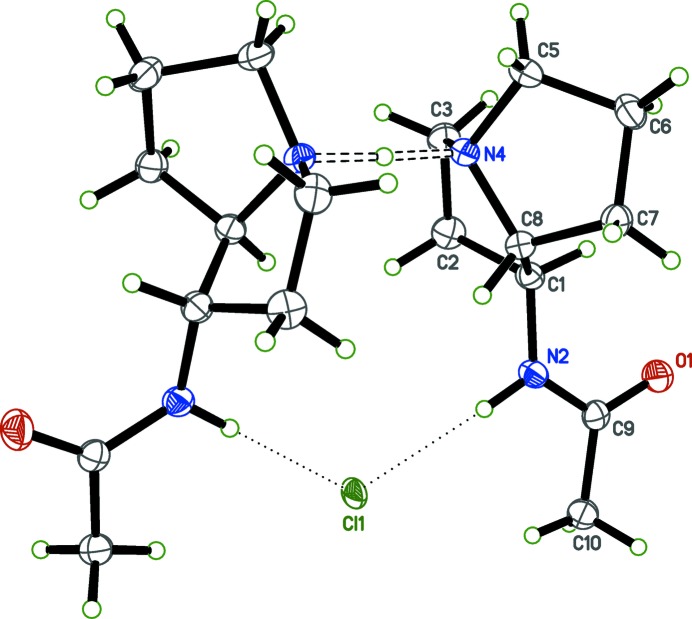
An ellipsoid plot (50% probability) showing the mol­ecular structure of 2AcAP·HCl. Unlabeled atoms are related to their labeled counterparts by a crystallographic twofold rotation (−*x* + 1, *y*, −*z* + 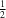). The unusually short N—H⋯N hydrogen bond between the pyrrolizidine N atoms is highlighted by open dashed lines. Weaker N—H⋯Cl hydrogen bonds between the acetamide NH group and the Cl^−^ anion are shown as dotted lines.

**Figure 3:**
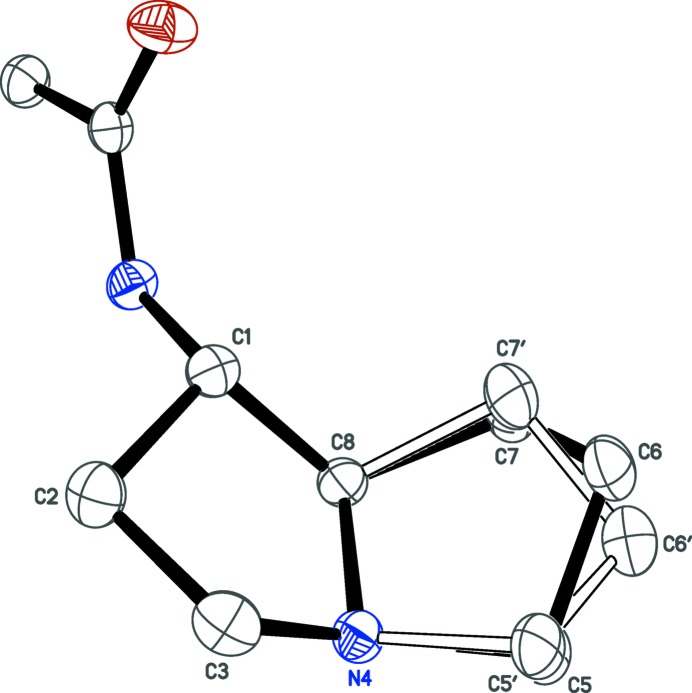
An ellipsoid plot (50% probability) showing disorder of the non-substituted half of the pyrrolizidine ring. The refined major:minor component occupancy factors are 0.897 (5):0.103 (5).

The mol­ecular structure of AcAP recrystallized from CHCl_3_ is a hemi-hydro­chloride, 2AcAP·HCl (Fig. 2 ▸). Within the pyrrolizidine group, the unsubstituted five-membered ring is disordered over two orientations (Fig. 3 ▸). Each five-membered ring has an envelope conformation, but since the minor component occupancy is small [*i.e*., 0.103 (5) *versus* 0.897 (5)], we restrict further comment to the major component only. The ‘flap’ atoms, C2 and C6, for the substituted (C1–C3/N4/C8) and unsubstituted (C5–C8/N4) rings, respectively, are displaced from the mean plane of the four remaining atoms (C1/C3/N4/C8 and C7/C5/N4/C8) by 0.622(1) and 0.633(2) Å, respectively. The dihedral angle between these mean planes is 56.07 (9)°. The acetamide group is characteristically almost planar (r.m.s. deviation = 0.0181 Å), but twisted from the mean plane of C1/C8/N4/C3 by a dihedral angle of 70.56 (7)°. Aside from an unusually short N—H⋯N hydrogen bond, which will be discussed in subsequent sections, all geometrical parameters are within their expected ranges (*e.g*., Allen *et al.*, 1987 ▸).

## Supra­molecular features   

**Figure 4:**
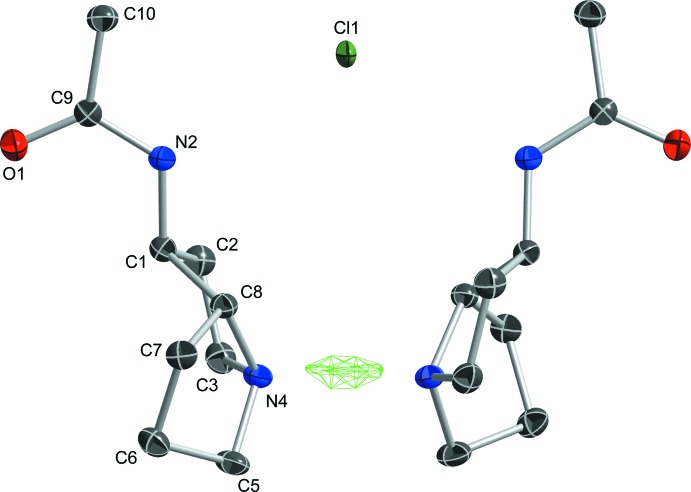
A difference-Fourier map showing elongated electron density for the hydrogen atom involved in the short N—H⋯N hydrogen bond of 2AcAP·HCl.

The primary structural motif within crystals of 2AcAP·HCl consists of a pair of homochiral AcAP mol­ecules hydrogen bonded to H^+^ and Cl^−^ ions about a crystallographic twofold axis of space group *C*2/*c*. Based on electron density alone, the H^+^ cation and Cl^−^ anion both *appear* to be located on the twofold axis. As a result of the twofold symmetry axis, the hydrogen bond N4—H4*N*⋯N4^i^ [symmetry code: (i) −*x* + 1, *y*, −*z* + 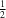] appears to be symmetric, and since *d*_N⋯N_ = 2.616 (2) Å (Table 1 ▸), it is exceptionally short (see *Database survey* section, below). Similarly, since the refined H^+^ position lies on the twofold axis and is equidistant from N4 and N4^i^, the *refined* N⋯H inter­atomic distance appears unusually long at 1.3080 (12) Å. The whole hydrogen-bonded ensemble makes an 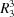
(12) supra­molecular motif. For the short N4—H4*N*⋯N4^i^ hydrogen bond, a difference-Fourier map (Fig. 4 ▸) clearly shows an elongated region of electron density centered on the twofold axis corresponding to the position of the refined H4*N* hydrogen atom. The true location of H4*N*, however, remains ambiguous based on X-ray data alone. Possibilities include strictly symmetric (*i.e*., on the twofold axis exactly halfway between N4 and N4^i^), statically disordered (*i.e*., 50% on each of N4 and N4^i^) or dynamically disordered (*i.e.*, exchanging rapidly between N4 and N4^i^). Alternative strategies for H4*N* inclusion in the model refined equally well (see *Refinement*, below), so we settled on the simplest approach, following the recommendations of Fábry (2018 ▸). Nonetheless, the ambiguity prompted us to analyze the potential energy surface of the H4*N* position *via* computational methods for single *versus* double energy well character (see *Computational analysis*, below). In addition to the strong N—H⋯N hydrogen bond, weaker N2—H2—Cl1—H2^i^—N2^i^ inter­actions link the twofold-related acetamide groups to the Cl^−^ anion [N⋯Cl = 3.2263 (11) Å]. The twist of the twofold-related hydrogen-bonded pyrrolizidine moieties relative to each other, as defined by the torsion angle C8—N4⋯N4^i^—C8^i^ is −69.82 (16)°. The almost planar acetamide group forms a dihedral angle with its twofold-related counterpart of 20.18 (3)°. The only other intra­molecular inter­actions are van der Waals contacts. Estimates of the relative fractions of inter­molecular contacts between individual atom types derived from a Hirshfeld-surface analysis using *CrystalExplorer* (Turner *et al.*, 2017 ▸; Tan *et al.*, 2019 ▸) are complicated by the ring disorder and by the N4—H4*N*⋯N4^i^ hydrogen bond. Nevertheless, all contacts appear to involve hydrogen atoms, with the overwhelming majority being H⋯H (65.3%). The Cl^−^ anion and the acetamide O atom each reside in pockets surrounded by hydrogen atoms, giving H⋯Cl/Cl⋯H (16.2%) and H⋯O/O⋯H (12.4%), with the remainder being N⋯H/H⋯N and C⋯H/H⋯C contacts.

## Database survey   

A search of the Cambridge Structure Database (version 5.40, Nov. 2018; Groom *et al.*, 2016 ▸) on the bicyclic pyrrolizidine core of AcAP yielded 584 hits. Of these, 41 are protonated at the ring N atom, but only three of those bear a substituent (other than H) at the 1-position of the pyrrolizidine ring system (assuming standard IUPAC ring numbering). CSD entry BRPYLZ (Wilson & Sawicki, 1979 ▸) is a bromide salt of a bromine derivative, and CPYRZD (Soderberg, 1971 ▸) is a zwitterion, with a carboxyl­ate group at the 1-position and a bromo substituent at the 2-position. The relative stereochemistry of BRPYLZ and CPYRZD, however, are different from AcAP. CSD entry EDOTUP (Bhardwaj *et al.*, 2017 ▸) was a precursor to AcAP, and is the most closely related currently deposited structure.

The most striking feature of 2AcAP·HCl is the unusually short N—H⋯N hydrogen bond. A CSD search on the fragment ‘C-N(*X*)-H-N(*X*)-C′, where ‘*X*′ denotes ‘any group’, gave 45 hits. Rejection of cases where apparent close N⋯N distances were due to disorder (four entries), and those in which the N-bound H atom was missing from the model (two entries), left 39 structures, of which three were duplicates. In the remaining 36 structures the N—H⋯N hydrogen bonds are *intra*mol­ecular in 22 and *inter*mol­ecular in 14. The closest N⋯N separations occur in the *intra*mol­ecular N—H⋯N hydrogen-bonded structures, the shortest being 2.419 Å in EBOKOV (Wilkes *et al.*, 2000 ▸). However, in these intra­molecular cases, the N⋯N separation is largely dictated by the intra­molecular geometry, which effectively forces the donor and acceptor N atoms into close proximity. Of the 14 CSD entries from the search that have *inter*mol­ecular N—H⋯N hydrogen bonding, the closest N⋯N separation occurs in BECHOG (Glidewell & Holden, 1982 ▸), in which a bis­(4-methyl­pyridine)­hydrogen(I) cation sits on an inversion centre, giving an apparently symmetric N—H⋯N hydrogen bond with N⋯N distance of 2.610 (15) Å, and ROHTIR (Bock *et al.*, 1997 ▸), for which the asymmetric unit contains two separate halves of a methyl­ammonium-methyl­amine cation, [CH_3_NH_2_-H-NH_2_CH_3_]^+^, each sitting on centres of inversion, giving N⋯N distances of 2.620 and 2.641 Å. The N⋯N separation in 2AcAP·HCl is similar, at 2.616 (2) Å, although the difference is not significant, and well within the quoted precision estimate of BECHOG and the accuracy limits imposed by the spherical-atom scattering-factor approximation (see *e.g.*, Dawson, 1964 ▸).

## Computational analysis   

**Figure 5:**
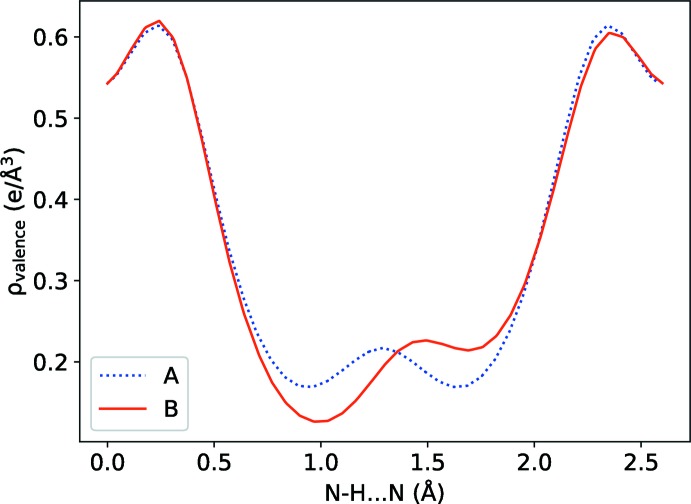
Computed charge-density line profile from nitro­gen to nitro­gen in the N—H⋯N hydrogen bond.

**Figure 6:**
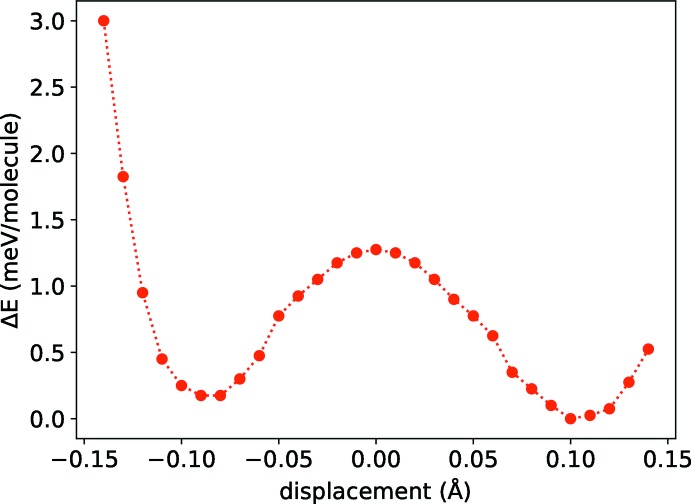
Computed potential-energy surface as a function of N—H⋯N hydrogen-atom displacement from the midpoint of the two nitro­gen atoms.

A model consisting of the four 2AcAP·HCl units present in one unit cell was relaxed at the DFT level (see below for details), both with and without symmetry constraints on charge density. In the case where symmetry constraints were absent, a small displacement (0.06 Å) was applied to the hydrogen atom in the N—H⋯N hydrogen bond to break symmetry in the initial geometry. The relaxations led to two structures, one with constrained twofold symmetry (*A*), and one unconstrained (*B*). The volume difference between these theoretical models (calculations assumed absolute zero) was negligible (*A*_vol_ = 1918.85 Å^3^ *versus B*_vol_ = 1919.63 Å^3^). In the symmetric model, the N—H⋯N hydrogen atom (corresponding to H4 in the crystallographic model) is equidistant between the two nitro­gen atoms (N—H = 1.290 Å), whereas in model *B* the N—H distances differ (N—H = 1.194 and 1.406 Å). This is in agreement with the computed charge-density line profile of N—H⋯N in structure *B*, as shown in Fig. 5 ▸. Structure *B* is calculated to be slightly more stable, but the energy difference (4.7 meV per unit cell) is vanishingly small (Fig. 6 ▸). The low energy barrier suggests dynamic disorder of the N—H⋯N hydrogen atom.

Computational details: For this periodic system, density functional theory (DFT) calculations were carried out using the Vienna *ab initio* simulation Package (VASP) with Perdew–Burke–Ernzerhof (PBE) exchange-correlation functional (Kresse & Furthmüller, 1996*a* ▸,*b* ▸; Kresse *et al.*, 1994 ▸; Perdew *et al.*, 1996 ▸). The electron-ion inter­actions were described with the projector augmented-wave (PAW) method (Blöchl, 1994 ▸; Kresse & Joubert, 1999 ▸). The valence electronic wavefunctions were expanded on a plane-wave basis with a kinetic energy cutoff at 520 eV, and Gaussian smearing with a width of 0.05 eV was employed. The convergence criterion of the total energy was set to 10 ^−5^ eV in the self-consistent field loop. The Brillouin zone was sampled with a 1×2×2 Γ-centered grid. The experimental structures were relaxed until the Hellman–Feynman forces for each site were less than 0.005 eV Å^−1^, and Grimme’s DFT-D3 dispersion correction was applied with Becke–Johnson damping (Grimme *et al.*, 2010 ▸, 2011 ▸).

## Synthesis and crystallization   

AcAP was synthesized and purified according to the published procedure (Pan *et al.*, 2014 ▸). Crystals of 2AcAP·HCl were obtained in the form of colorless plates by dissolving 20 mg of AcAP in 1 ml of CHCl_3_ in a 10 ml round-bottom flask and allowing the solution to stand in a refrigerator for about a month.

## Refinement   

Crystal data, data collection, and structure refinement details are given in Table 2 ▸. Non-disordered carbon-bound H atoms were found in difference-Fourier maps, but subsequently included in the refinement using riding models, with constrained distances set to 0.98 Å (*R*CH_3_), 0.99 Å (*R*_2_CH_2_) and 1.00 Å (*R*_3_CH). Following the advice of Fábry (2018 ▸), the hydrogen atom involved in the short N—H⋯N hydrogen bond (H4*N*) was placed into difference-Fourier electron density and refined, albeit constrained to the twofold axis. An alternative model in which this H atom was allowed to ride at 50% occupancy on both N4 and N4^i^ [symmetry code: (i) −*x* + 1, *y*, −*z* + 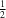] refined equally well: the X-ray data alone being insufficient to establish a preference. The amide N—H hydrogen atom (H2*N*) was refined freely. *U*_iso_(H) parameters for nitro­gen-bound hydrogen atoms were refined, while for carbon-bound H atoms, *U*_iso_(H) were set to values of either 1.2*U*_eq_ (*R*_3_CH, *R*_2_H_2_) or 1.5*U*_eq_ (*R*CH_3_) of the attached atom.

The refined displacement parameters for the Cl^−^ anion (*e.g.*, Figs. 2 ▸ and 4 ▸) appear a little small compared to the rest of the structure. In addition, the largest residual difference map peaks (0.67 and 0.65 e Å^−3^) were close (0.37 and 0.47 Å respectively) to Cl1. Refinement of the anion as mixed Cl and Br gave an occupancy ratio of 0.934 (2):0.066 (2), a lower *R*-value (3.02%), and a flatter difference map (Δρ = 0.29/-0.19 e Å^−3^). However, the reaction included no known source of Br^−^, so the mixed anion model was not retained.

To ensure satisfactory refinement for disordered groups in the structure, a combination of constraints and restraints were employed. The constraints (*SHELXL* commands EXYZ and EADP) were used to fix overlapping fragments. Restraints were used to ensure the integrity of ill-defined or disordered groups (*SHELXL* commands SAME, SIMU, and RIGU). An alternative model using space group *Cc* (50:50 inversion twinned) was considered but rejected as it required hefty restraints and did not resolve the H4*N* atom ambiguity.

## Supplementary Material

Crystal structure: contains datablock(s) I. DOI: 10.1107/S2056989019016517/hb7870sup1.cif

Structure factors: contains datablock(s) I. DOI: 10.1107/S2056989019016517/hb7870Isup2.hkl

CCDC reference: 1970639

Additional supporting information:  crystallographic information; 3D view; checkCIF report

## Figures and Tables

**Table 1:** Hydrogen-bond geometry (Å, °)

*D*—H⋯*A*	*D*—H	H⋯*A*	*D*⋯*A*	*D*—H⋯*A*
N4—H4*N*⋯N4^i^	1.31 (1)	1.31 (1)	2.616 (2)	178 (3)
N2—H2*N*⋯Cl1	0.94 (2)	2.29 (2)	3.2263 (11)	176.4 (16)

Symmetry code: (i) 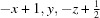.

**Table 2:** Experimental details

Crystal data	
Chemical formula	C_9_H_17_N_2_O^+^·Cl^−^·C_9_H_16_N_2_O
*M*_r_	372.93
Crystal system, space group	Monoclinic, *C*2/*c*
Temperature (K)	90
*a*, *b*, *c* (Å)	20.2125 (8), 9.6926 (4), 10.1458 (3)
β (°)	100.445 (1)
*V* (Å^3^)	1954.74 (13)
*Z*	4
Radiation type	Mo *K*α
μ (mm^−1^)	0.22
Crystal size (mm)	0.40 × 0.36 × 0.04

Data collection	
Diffractometer	Bruker D8 Venture dual source
Absorption correction	Multi-scan (*SADABS*; Krause *et al.*, 2015 ▸)
*T*_min_, *T*_max_	0.885, 0.988
No. of measured, independent and observed [*I* > 2σ(*I*)] reflections	31903, 2235, 2096
*R*_int_	0.032
(sin θ/λ)_max_ (Å^−1^)	0.649

Refinement	
*R*[*F* ^2^ > 2σ(*F* ^2^)], *wR*(*F* ^2^), *S*	0.037, 0.096, 1.12
No. of reflections	2235
No. of parameters	144
No. of restraints	82
H-atom treatment	H atoms treated by a mixture of independent and constrained refinement
Δρ_max_, Δρ_min_ (e Å^−3^)	0.67, −0.22

Computer programs: *APEX3* (Bruker, 2016 ▸), *SHELXT* (Sheldrick, 2015*a* ▸), *SHELXL2018/3* (Sheldrick, 2015*b* ▸), *XP* in *SHELXTL* and *SHELX* (Sheldrick, 2008 ▸), *CIFFIX* (Parkin, 2013 ▸) and *PLATON* (Spek, 2009 ▸).

## supplementary crystallographic information

### Crystal data

**Table:**

C_9_H_17_N_2_O^+^·Cl^−^·C_9_H_16_N_2_O	*F*(000) = 808
*M**_r_* = 372.93	*D*_x_ = 1.267 Mg m^−^^3^
Monoclinic, *C*2/*c*	Mo *K*α radiation, λ = 0.71073 Å
*a* = 20.2125 (8) Å	Cell parameters from 9978 reflections
*b* = 9.6926 (4) Å	θ = 2.3–27.5°
*c* = 10.1458 (3) Å	µ = 0.22 mm^−^^1^
β = 100.445 (1)°	*T* = 90 K
*V* = 1954.74 (13) Å^3^	Plate, colourless
*Z* = 4	0.40 × 0.36 × 0.04 mm

### Data collection

**Table:**

Bruker D8 Venture dual source diffractometer	2235 independent reflections
Radiation source: microsource	2096 reflections with *I* > 2σ(*I*)
Detector resolution: 7.41 pixels mm^-1^	*R*_int_ = 0.032
φ and ω scans	θ_max_ = 27.5°, θ_min_ = 2.3°
Absorption correction: multi-scan (*SADABS*; Krause *et al.*, 2015)	*h* = −26→26
*T*_min_ = 0.885, *T*_max_ = 0.988	*k* = −12→12
31903 measured reflections	*l* = −11→13

### Refinement

**Table:**

Refinement on *F*^2^	Secondary atom site location: difference Fourier map
Least-squares matrix: full	Hydrogen site location: mixed
*R*[*F*^2^ > 2σ(*F*^2^)] = 0.037	H atoms treated by a mixture of independent and constrained refinement
*wR*(*F*^2^) = 0.096	*w* = 1/[σ^2^(*F*_o_^2^) + (0.0375*P*)^2^ + 2.4571*P*] where *P* = (*F*_o_^2^ + 2*F*_c_^2^)/3
*S* = 1.12	(Δ/σ)_max_ < 0.001
2235 reflections	Δρ_max_ = 0.67 e Å^−^^3^
144 parameters	Δρ_min_ = −0.22 e Å^−^^3^
82 restraints	Extinction correction: SHELXL-2018/3 (Sheldrick 2018), Fc^*^=kFc[1+0.001xFc^2^λ^3^/sin(2θ)]^-1/4^
Primary atom site location: structure-invariant direct methods	Extinction coefficient: 0.005 (1)

### Special details

**Table:**

Experimental. The crystal was mounted using polyisobutene oil on the tip of a fine glass fibre, which was fastened in a copper mounting pin with electrical solder. It was placed directly into the cold gas stream of a liquid-nitrogen based cryostat (Hope, 1994; Parkin & Hope, 1998). Diffraction data were collected with the crystal at 90K, which is standard practice in this laboratory for the majority of flash-cooled crystals.
Geometry. All esds (except the esd in the dihedral angle between two l.s. planes) are estimated using the full covariance matrix. The cell esds are taken into account individually in the estimation of esds in distances, angles and torsion angles; correlations between esds in cell parameters are only used when they are defined by crystal symmetry. An approximate (isotropic) treatment of cell esds is used for estimating esds involving l.s. planes.
Refinement. Refinement progress was checked using *PLATON* (Spek, 2009) and by an *R*-tensor (Parkin, 2000). The final model was further checked with the IUCr utility *checkCIF*.

### Fractional atomic coordinates and isotropic or equivalent isotropic displacement parameters (Å^2^)

**Table:**

	*x*	*y*	*z*	*U*_iso_*/*U*_eq_	Occ. (<1)
C1	0.63783 (6)	0.58377 (14)	0.26083 (13)	0.0182 (3)	
H1	0.684523	0.622089	0.275591	0.022*	
C2	0.60256 (7)	0.61745 (15)	0.11816 (14)	0.0229 (3)	
H2A	0.632627	0.601397	0.052759	0.027*	
H2B	0.561093	0.562062	0.092144	0.027*	
C3	0.58652 (7)	0.76915 (15)	0.12835 (14)	0.0232 (3)	
H3A	0.626574	0.826636	0.124182	0.028*	
H3B	0.549608	0.797166	0.055229	0.028*	
N4	0.56555 (6)	0.78256 (11)	0.26254 (11)	0.0184 (3)	
H4N	0.500000	0.781 (3)	0.250000	0.056 (9)*	
C5	0.5885 (2)	0.9121 (2)	0.3373 (3)	0.0249 (7)	0.897 (5)
H5A	0.554374	0.945176	0.388570	0.030*	0.897 (5)
H5B	0.597261	0.985720	0.275018	0.030*	0.897 (5)
C6	0.65299 (9)	0.87111 (17)	0.43106 (16)	0.0237 (4)	0.897 (5)
H6A	0.663640	0.935711	0.507555	0.028*	0.897 (5)
H6B	0.691530	0.867102	0.383296	0.028*	0.897 (5)
C7	0.63499 (12)	0.7276 (2)	0.4771 (2)	0.0209 (5)	0.897 (5)
H7A	0.675941	0.673734	0.512861	0.025*	0.897 (5)
H7B	0.606530	0.733983	0.546696	0.025*	0.897 (5)
C8	0.59630 (6)	0.66296 (13)	0.34850 (13)	0.0178 (3)	0.897 (5)
H8	0.559801	0.602145	0.370299	0.021*	0.897 (5)
C5'	0.595 (2)	0.9165 (19)	0.322 (2)	0.0249 (7)	0.103 (5)
H5'A	0.561158	0.991410	0.308580	0.030*	0.103 (5)
H5'B	0.634307	0.944647	0.282171	0.030*	0.103 (5)
C6'	0.6157 (9)	0.8793 (15)	0.4690 (15)	0.032 (3)	0.103 (5)
H6'A	0.576222	0.871718	0.513823	0.039*	0.103 (5)
H6'B	0.647840	0.947113	0.517395	0.039*	0.103 (5)
C7'	0.6489 (11)	0.739 (2)	0.458 (2)	0.030 (4)	0.103 (5)
H7'A	0.655595	0.689122	0.544614	0.036*	0.103 (5)
H7'B	0.692818	0.749314	0.428958	0.036*	0.103 (5)
C8'	0.59630 (6)	0.66296 (13)	0.34850 (13)	0.0178 (3)	0.103 (5)
H8'	0.563026	0.605013	0.385455	0.021*	0.103 (5)
N2	0.64091 (6)	0.43798 (12)	0.29157 (11)	0.0184 (3)	
H2N	0.6002 (10)	0.390 (2)	0.2837 (19)	0.035 (5)*	
C9	0.69841 (6)	0.36514 (14)	0.31606 (12)	0.0178 (3)	
O1	0.75448 (5)	0.41694 (11)	0.32142 (11)	0.0260 (2)	
C10	0.68897 (7)	0.21311 (14)	0.33748 (14)	0.0224 (3)	
H10A	0.715908	0.185291	0.423611	0.034*	
H10B	0.703494	0.160954	0.265024	0.034*	
H10C	0.641361	0.194212	0.337727	0.034*	
Cl1	0.500000	0.27309 (5)	0.250000	0.02141 (15)	

### Atomic displacement parameters (Å^2^)

**Table:**

	*U*^11^	*U*^22^	*U*^33^	*U*^12^	*U*^13^	*U*^23^
C1	0.0169 (6)	0.0170 (6)	0.0210 (6)	−0.0016 (5)	0.0044 (5)	0.0007 (5)
C2	0.0266 (7)	0.0247 (7)	0.0185 (6)	−0.0007 (5)	0.0071 (5)	0.0009 (5)
C3	0.0240 (7)	0.0244 (7)	0.0214 (7)	−0.0021 (5)	0.0041 (5)	0.0056 (5)
N4	0.0201 (5)	0.0139 (5)	0.0201 (6)	−0.0016 (4)	0.0006 (4)	0.0003 (4)
C5	0.0284 (13)	0.0147 (7)	0.0291 (10)	−0.0044 (6)	−0.0014 (9)	−0.0013 (6)
C6	0.0240 (9)	0.0220 (8)	0.0234 (8)	−0.0059 (6)	0.0001 (6)	−0.0025 (6)
C7	0.0237 (10)	0.0206 (8)	0.0174 (9)	0.0000 (7)	0.0015 (7)	−0.0004 (7)
C8	0.0189 (6)	0.0154 (6)	0.0187 (6)	0.0002 (5)	0.0020 (5)	0.0012 (5)
C5'	0.0284 (13)	0.0147 (7)	0.0291 (10)	−0.0044 (6)	−0.0014 (9)	−0.0013 (6)
C6'	0.033 (7)	0.028 (5)	0.033 (5)	−0.003 (5)	−0.001 (5)	−0.008 (4)
C7'	0.034 (8)	0.034 (6)	0.017 (7)	0.002 (6)	−0.003 (6)	−0.006 (5)
C8'	0.0189 (6)	0.0154 (6)	0.0187 (6)	0.0002 (5)	0.0020 (5)	0.0012 (5)
N2	0.0175 (5)	0.0155 (6)	0.0228 (6)	−0.0015 (4)	0.0049 (4)	−0.0005 (4)
C9	0.0200 (6)	0.0191 (6)	0.0142 (6)	−0.0007 (5)	0.0028 (5)	−0.0010 (5)
O1	0.0179 (5)	0.0247 (5)	0.0349 (6)	−0.0010 (4)	0.0029 (4)	0.0037 (4)
C10	0.0246 (7)	0.0178 (7)	0.0239 (7)	0.0004 (5)	0.0022 (5)	−0.0015 (5)
Cl1	0.0099 (2)	0.0177 (2)	0.0354 (3)	0.000	0.00096 (16)	0.000

### Geometric parameters (Å, º)

**Table:**

C1—N2	1.4460 (17)	C6—H6B	0.9900
C1—C2	1.5284 (19)	C7—C8	1.529 (2)
C1—C8	1.5347 (18)	C7—H7A	0.9900
C1—C8'	1.5347 (18)	C7—H7B	0.9900
C1—H1	1.0000	C8—H8	1.0000
C2—C3	1.513 (2)	C5'—C6'	1.521 (18)
C2—H2A	0.9900	C5'—H5'A	0.9900
C2—H2B	0.9900	C5'—H5'B	0.9900
C3—N4	1.5032 (18)	C6'—C7'	1.528 (17)
C3—H3A	0.9900	C6'—H6'A	0.9900
C3—H3B	0.9900	C6'—H6'B	0.9900
N4—H4N	1.3080 (12)	C7'—C8'	1.576 (16)
N4—C5	1.497 (2)	C7'—H7'A	0.9900
N4—C5'	1.507 (17)	C7'—H7'B	0.9900
N4—C8	1.5146 (16)	C8'—H8'	1.0000
N4—C8'	1.5146 (16)	N2—C9	1.3439 (17)
N4—H4N	1.3080 (12)	N2—H2N	0.94 (2)
C5—C6	1.520 (4)	C9—O1	1.2317 (16)
C5—H5A	0.9900	C9—C10	1.5066 (19)
C5—H5B	0.9900	C10—H10A	0.9800
C6—C7	1.532 (3)	C10—H10B	0.9800
C6—H6A	0.9900	C10—H10C	0.9800
			
N2—C1—C2	114.05 (11)	C8—C7—C6	102.97 (16)
N2—C1—C8	111.75 (11)	C8—C7—H7A	111.2
C2—C1—C8	103.41 (11)	C6—C7—H7A	111.2
N2—C1—C8'	111.75 (11)	C8—C7—H7B	111.2
C2—C1—C8'	103.41 (11)	C6—C7—H7B	111.2
N2—C1—H1	109.1	H7A—C7—H7B	109.1
C2—C1—H1	109.1	N4—C8—C7	105.73 (12)
C8—C1—H1	109.1	N4—C8—C1	105.05 (10)
C3—C2—C1	102.22 (11)	C7—C8—C1	116.80 (13)
C3—C2—H2A	111.3	N4—C8—H8	109.7
C1—C2—H2A	111.3	C7—C8—H8	109.7
C3—C2—H2B	111.3	C1—C8—H8	109.7
C1—C2—H2B	111.3	N4—C5'—C6'	101.8 (12)
H2A—C2—H2B	109.2	N4—C5'—H5'A	111.4
N4—C3—C2	104.18 (11)	C6'—C5'—H5'A	111.4
N4—C3—H3A	110.9	N4—C5'—H5'B	111.4
C2—C3—H3A	110.9	C6'—C5'—H5'B	111.4
N4—C3—H3B	110.9	H5'A—C5'—H5'B	109.3
C2—C3—H3B	110.9	C5'—C6'—C7'	100.7 (16)
H3A—C3—H3B	108.9	C5'—C6'—H6'A	111.6
H4N—N4—C5	106.1 (12)	C7'—C6'—H6'A	111.6
H4N—N4—C3	110.96 (17)	C5'—C6'—H6'B	111.6
C5—N4—C3	114.78 (16)	C7'—C6'—H6'B	111.6
H4N—N4—C5'	112 (2)	H6'A—C6'—H6'B	109.4
C3—N4—C5'	106.2 (15)	C6'—C7'—C8'	102.6 (11)
H4N—N4—C8	110.4 (12)	C6'—C7'—H7'A	111.2
C5—N4—C8	107.04 (14)	C8'—C7'—H7'A	111.2
C3—N4—C8	107.55 (10)	C6'—C7'—H7'B	111.2
H4N—N4—C8'	110.4 (12)	C8'—C7'—H7'B	111.2
C3—N4—C8'	107.55 (10)	H7'A—C7'—H7'B	109.2
C5'—N4—C8'	109.8 (9)	N4—C8'—C1	105.05 (10)
H4N—N4—H4N	0 (3)	N4—C8'—C7'	101.6 (8)
C5—N4—H4N	106.1 (12)	C1—C8'—C7'	105.9 (9)
C3—N4—H4N	110.96 (17)	N4—C8'—H8'	114.3
C5'—N4—H4N	112 (2)	C1—C8'—H8'	114.3
C8—N4—H4N	110.4 (12)	C7'—C8'—H8'	114.3
C8'—N4—H4N	110.4 (12)	C9—N2—C1	123.73 (11)
N4—C5—C6	104.5 (2)	C9—N2—H2N	118.0 (12)
N4—C5—H5A	110.9	C1—N2—H2N	117.9 (12)
C6—C5—H5A	110.9	O1—C9—N2	123.50 (13)
N4—C5—H5B	110.9	O1—C9—C10	122.14 (13)
C6—C5—H5B	110.9	N2—C9—C10	114.37 (12)
H5A—C5—H5B	108.9	C9—C10—H10A	109.5
C5—C6—C7	101.78 (16)	C9—C10—H10B	109.5
C5—C6—H6A	111.4	H10A—C10—H10B	109.5
C7—C6—H6A	111.4	C9—C10—H10C	109.5
C5—C6—H6B	111.4	H10A—C10—H10C	109.5
C7—C6—H6B	111.4	H10B—C10—H10C	109.5
H6A—C6—H6B	109.3		
			
N2—C1—C2—C3	162.24 (11)	N2—C1—C8—C7	93.71 (16)
C8—C1—C2—C3	40.67 (13)	C2—C1—C8—C7	−143.19 (14)
C8'—C1—C2—C3	40.67 (13)	H4N—N4—C5'—C6'	97 (2)
C1—C2—C3—N4	−39.46 (13)	C3—N4—C5'—C6'	−142 (2)
C2—C3—N4—H4N	−97.5 (15)	C8'—N4—C5'—C6'	−26 (3)
C2—C3—N4—C5	142.30 (19)	N4—C5'—C6'—C7'	45 (3)
C2—C3—N4—C5'	140.9 (15)	C5'—C6'—C7'—C8'	−47 (2)
C2—C3—N4—C8	23.32 (13)	H4N—N4—C8'—C1	123.3 (9)
C2—C3—N4—C8'	23.32 (13)	C3—N4—C8'—C1	2.12 (13)
H4N—N4—C5—C6	141.3 (8)	C5'—N4—C8'—C1	−113 (2)
C3—N4—C5—C6	−95.81 (19)	H4N—N4—C8'—C7'	−126.5 (13)
C8—N4—C5—C6	23.5 (3)	C3—N4—C8'—C7'	112.3 (10)
N4—C5—C6—C7	−39.9 (3)	C5'—N4—C8'—C7'	−3 (2)
C5—C6—C7—C8	40.9 (2)	N2—C1—C8'—N4	−149.53 (11)
H4N—N4—C8—C7	−112.6 (9)	C2—C1—C8'—N4	−26.43 (13)
C5—N4—C8—C7	2.4 (2)	N2—C1—C8'—C7'	103.4 (9)
C3—N4—C8—C7	126.23 (14)	C2—C1—C8'—C7'	−133.5 (9)
H4N—N4—C8—C1	123.3 (9)	C6'—C7'—C8'—N4	30.6 (18)
C5—N4—C8—C1	−121.70 (19)	C6'—C7'—C8'—C1	140.1 (14)
C3—N4—C8—C1	2.12 (13)	C2—C1—N2—C9	113.29 (14)
C6—C7—C8—N4	−26.97 (19)	C8—C1—N2—C9	−129.88 (13)
C6—C7—C8—C1	89.41 (17)	C8'—C1—N2—C9	−129.88 (13)
N2—C1—C8—N4	−149.53 (11)	C1—N2—C9—O1	3.8 (2)
C2—C1—C8—N4	−26.43 (13)	C1—N2—C9—C10	−176.47 (12)

### Hydrogen-bond geometry (Å, º)

**Table:**

*D*—H···*A*	*D*—H	H···*A*	*D*···*A*	*D*—H···*A*
N4—H4*N*···N4^i^	1.31 (1)	1.31 (1)	2.616 (2)	178 (3)
N2—H2*N*···Cl1	0.94 (2)	2.29 (2)	3.2263 (11)	176.4 (16)

Symmetry code: (i) −*x*+1, *y*, −*z*+1/2.
